# Exploring different definitions of methane phenotypes in Dutch Holstein cattle

**DOI:** 10.3168/jdsc.2025-0845

**Published:** 2025-10-10

**Authors:** C.I.V. Manzanilla-Pech, A.E. van Breukelen, R.F. Veerkamp, Y. de Haas, M. van Pelt, B. Gredler-Grandl

**Affiliations:** 1 Animal Breeding and Genomics, Wageningen Livestock Research, Wageningen University & Research, 6708 PB Wageningen, the Netherlands; 2 Cooperation CRV, Animal Evaluation Unit, 6800 AL Arnhem, Netherlands

## Abstract

One of the most promising strategies to permanently reduce methane emissions in dairy cattle is through genomic selection, where the primary goal is to identify and selectively breed low-emitting ruminants. An important step is to define which trait definition to use. Several methane phenotypes have been proposed in recent years. When measuring methane using breath analyzers (sniffers), 2 commonly used phenotypes are methane concentration (CH_4_c; ppm) and methane production (CH_4_p; g/d). However, different definitions exist for both phenotypes. For example, for CH_4_c, the average per visit is commonly used, but other phenotypes involving the identification of eructation peaks have been suggested. Several formulas are available to calculate CH_4_p, using CH_4_c or the ratio between CH_4_c and CO_2_ concentrations as input, together with other production traits. Additionally, methane intensity (MeI), another phenotype of interest, is the amount of methane produced per milk unit (kg). Therefore, the aims of this study were (1) to estimate genetic parameters for 11 distinct phenotypes, including 3 CH_4_c definitions, carbon dioxide concentration per visit (CO_2_c), the ratio between CH_4_c and CO_2_c, 5 phenotypes for CH_4_p based on different formulas (Madsen, Chagunda, 2 of Kjeldsen, and IPCC Tier2 as a benchmark), and MeI; and (2) to estimate genetic correlations between these methane phenotypes and milk yield (MY) and BW. A total of 149,726 sniffer (CH_4_c and CO_2_c) records were available from 7,600 Dutch Holstein cows measured between 2019 and 2024 on 68 farms. Data were analyzed with an animal repeatability model with fixed effects, including herd-year-season, week of lactation, and lactation number with age of cow at calving nested. Estimated heritability values ranged from 0.16 to 0.30 for CH_4_c phenotypes, with the number of eructation peaks having the highest heritability. For CH_4_p, heritabilities ranged from 0.03 to 0.27, with Tier2 having the highest value. Heritabilities for the CH_4_c/CO_2_c ratio and CO_2_c were 0.08 and 0.13, respectively. Genetic correlations between CH_4_c phenotypes were moderate to highly positive, ranging between 0.49 and 0.85. Likewise, highly positive genetic correlations (between 0.89 and 1) were estimated for CH_4_p phenotypes, except for Tier2, which presented correlations between 0.04 and 0.37 with the other CH_4_p phenotypes. Average CH_4_c (avg) per visit and sum of maximum peaks of CH_4_c (speaks) had moderate to high positive correlations (0.36–0.95) with the majority of the CH_4_p phenotypes (except Tier2). Methane intensity had moderate to high positive genetic correlations (0.38 to 0.80) with the majority of the phenotypes except for Kjeldsen2 (0.03) and Tier2 (−0.90). Milk yield had positive genetic correlations with all the methane phenotypes (0.04 to 0.94) except for MeI (−0.66) and Kjeldsen2 (−0.69). Body weight had close to zero genetic correlations with CH_4_c phenotypes (−0.09 to 0.07), and a moderate positive to moderate negative correlation (−0.72 to 0.57) for CH_4_p phenotypes. Given their strong correlations with the other methane phenotypes, close to zero correlation with body weight, and no induced dependencies with BW and MY, as seen with the CH_4_p phenotypes, avg and speaks appear to suitable proxies for methane emissions when using sniffers.

## Highlights

• Average CH_4_c is positively correlated with most of the phenotypes except for ratio (CH_4_c and CO_2_ concentration).
• Methane production phenotypes derived by formulas are highly positively correlated among them.
• Methane intensity is positively correlated with most methane phenotypes.

## Graphical Abstract

**Figure:**
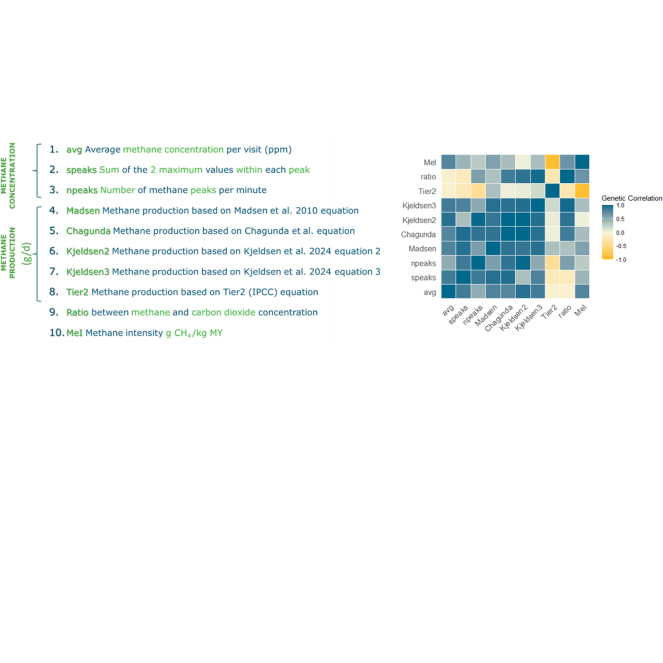
**Summary:** Several methane phenotypes have been proposed in recent years. When measuring methane using breath analyzers (sniffers), 2 commonly used phenotypes are methane concentration (CH4c; ppm) and methane production (CH_4_p; g/d). However, even within these phenotypes, different definitions exist. For example, for CH_4_c, the average per visit is commonly used, but other phenotypes involving the identification of “eructation” peaks have been proposed. For CH_4_p, there are several available formulas to calculate it, using methane concentration or ratio as input, together with other production traits. Additionally, methane intensity is another phenotype that has interest, as it represents the amount of methane per production unit (in dairy cattle, milk). Genetic correlations within each group of phenotypes (CH_4_c and CH_4_p) were highly positive, except for the formula of the Intergovernmental Panel on Climate Change (IPCC) to predict methane that was used as a benchmark (Tier2). Methane intensity had negative genetic correlations with the majority of the phenotypes.

Methane emissions in dairy cattle have been investigated intensively over the past decade due to the 2030–2050 climate targets, where the European Union aims for a 55% reduction up to neutrality in greenhouse gas emissions (EEA, 2023). One of the mitigation options is through genetic and genomic selection, where the primary goal is to identify and selectively breed low-emitting animals. As a result, several countries are currently monitoring enteric methane from ruminants. A widely employed phenotyping method involves breath sampling during milking by a breath analyzer device commonly referred to as a sniffer. This device samples breath at regular intervals (often between 1 and 15 s) during milking, reporting methane concentration (**CH_4_c**) in parts per million. Although CH_4_c has been proposed as an indicator for gross methane emissions (referred to as methane production [**CH_4_p**]; CH_4_ g/d) due to its high correlation (0.76; van Breukelen et al., 2023), there is a lack of consensus on which phenotype to use for estimating breeding values. Several phenotypes have been proposed over the past years; one that is largely used is the average CH_4_c per visit or per minute averaged daily or weekly, but other CH_4_c phenotypes involving the identification of eructation peaks have also been suggested (Garnsworthy et al., 2012; Rey et al., 2019; Reintke et al., 2020). Both studies that investigated peaks used CH_4_c measured by mobile laser methane detectors. Rey et al. (2019) defined the peaks as the number of peaks or eructation events presented in 5 min. Reintke et al. (2020) proposed the sum of CH_4_c per minute during eructation peaks, maximum CH_4_c during eructation peaks, and the number of eructation events per minute as possible CH_4_c phenotypes in ewe breeding. The rationale behind the use of eructation peaks as a phenotype is to try to disentangle CH_4_c that comes from a respiration event from the one that comes from an eructation event (i.e., the peaks). Previous studies by nutritionists (Blaxter and Joyce, 1963; Murray et al., 1976) showed that ∼17% of the methane exhaled originates from the lungs, and the remaining 83% is produced by eructation. Additionally, eructation peak detection could adjust for barn ambient CH_4_c, as well as for build-up of CH_4_c in the automated milking system (**AMS**) feed bin during milking (Bell, et al., 2014). Although no literature has been published on the heritability of eructation peaks in cows, several studies have reported the detection of methane peaks using sniffers (Rey et al., 2019; Hardan et al., 2022). Additionally, sniffers traditionally rely on formulas to convert CH_4_c (ppm) to CH_4_p (g/d). Currently, several formulas are available to calculate CH_4_p using CH_4_c and BW (Chagunda et al., 2009) or predicting carbon dioxide (CO_2_ g/d; via heat production using BW, ECM, and days in pregnancy) and multiplying it by the ratio between CH_4_c and CO_2_ concentration (**CO_2_c**) to predict CH_4_p (Pedersen et al., 2008; Madsen et al., 2010). Although some of these formulas are extensively used, they were originally developed to predict CH_4_p in barns, not individual animal CH_4_p. Recently, Kjeldsen et al. (2024) developed 3 formulas to predict CO_2_ (g/d) using an international dataset of more than 2,000 cows with recorded CH_4_p using either respiration chambers or GreenFeed units (C-Lock, Inc.). Finally, the IPCC (2019) has proposed several approaches (designated Tier1 to Tier3) to calculate methane emission factors per species, where the Tier2 formula is extensively used for dairy cattle, as it uses an energy balance approach. Therefore, the aims of this study were to (1) estimate genetic parameters for 11 distinct phenotypes, including 3 CH_4_c definitions, CO_2_c per visit, the ratio between average CH_4_c and CO_2_c, 5 phenotypes for CH_4_p based on different formulas (Madsen, Chagunda, Kjeldsen, and Tier2), and methane intensity (**MeI**); and (2) estimate genetic correlations between these methane phenotypes and milk yield (**MY**; kg) and BW.

The original data included a total of 149,726 sniffer CH_4_c and CO_2_c records from roughly 7,600 primi- and multiparous Dutch Holstein cows. The data have been previously described in van Breukelen et al. (2023, 2024). Methane concentration was measured using sniffers located in the AMS (WD-WUR v2.0, Carltech BV), sampling CH_4_c and CO_2_c every 5 s. Based on the sniffer CH_4_c and CO_2_c (ppm) measurements, 11 phenotypes were derived. We divided the phenotypes into groups: CH_4_c (in ppm), CH_4_p (in g/d), and MeI (in g CH_4_/kg MY). Methane concentration phenotypes included (1) the average CH_4_c per visit (**avg**), (2) the number of eructation peaks per minute (**npeaks**), and (3) the sum of maximum eructation peaks (**speaks**), defined as the sum of the average of the 2 top values within each peak (for all peaks within visit). Methane production phenotypes were calculated with 5 formulas available in the literature: (4) Madsen (Madsen et al., 2010), based on the prediction of CO_2_ using ECM and BW to posteriorly multiply it by ratio; (5) Chagunda (Chagunda et al., 2009), based on the tidal volume that uses BW as a predictor and the average CH_4_c; (6) and (7) Kjeldsen2 and Kjeldsen3 (Kjeldsen et al., 2024), based on the prediction of CO_2_ using ECM and BW or only ECM, and multiplied by the ratio between CH_4_c and CO_2_c; and (8) Tier2 (IPCC, 2019), based on the prediction of CH_4_p using MY and BW. Tier 2 was used as a benchmark scenario incorporating information on MY, BW, and diet, without relying on sniffer-based measurements, and was not treated as a selection phenotype. Additionally, we included as phenotypes (9) the ratio of CH_4_c to CO_2_c (**ratio**), (10) CO_2_c, and (11) MeI, defined as CH_4_p divided by MY (g CH_4_p/kg MY), where CH_4_p was calculated using the Kjeldsen3 formula (as it was the phenotype with the largest number of records and animals). Each of the 11 phenotypes was calculated at the visit level. Weekly CH_4_c and CH_4_p phenotypes were calculated by averaging visit phenotypes (with a minimum number of 5 visits) per calendar week. All CH_4_c phenotypes had a background level subtracted, where the background was calculated as the 5 lowest measurements during the entire visit, in order to account for the CH_4_c level in the barn. Furthermore, visit CH_4_c and CH_4_p were defined as the concentrations present in a window of 240 s (between 60 and 300 s after the entrance time of the cow in the AMS). The first 60 s were removed to account for the gas traveling through the tube until the gas sensor. Visits were cut at 300 s, and visits shorter than 300 s were discarded to avoid erratic data due to head movement, as shown by Løvendahl et al. (2024), as pellets are dropped in the AMS and consumed (mainly) in the first minutes of the milking. Eructation peaks were defined using R, detecting a local maxima by computing the first and the second differences of the signal (0.0005). The function calculates the change between consecutive values. A peak is defined as a point where the signal transitions from increasing to decreasing (i.e., where the first derivative is positive followed by a negative slope). Once eructation peaks were defined, 2 phenotypes were calculated: (1) the number of peaks per minute, which is the total number of peaks per visit divided by visit length, and (2) the 2 maximum values per peak, which were detected, averaged, and then summed across all the peaks per visit. The majority of animals had MY and milk content information, however, only ∼10% of the animals had BW. For this reason, phenotypes that used BW or metabolic BW (**MBW**) had a smaller number of records and animals. The number of records and animals per phenotype after editing is presented in Table 1.

**Table 1:** Descriptive statistics for the CH_4_c per visit (avg); number of peaks per minute (npeaks); sum of maximum peaks per minute (speaks); CH_4_p calculated by Madsen, Chagunda, Kjeldsen2, Kjeldsen3, and Tier2; ratio between CH_4_c and CO_2_c; carbon dioxide per visit (CO_2_c); methane intensity (MeI); and milk yield (MY) and BW

Trait	Cows (n)	Records (n)	Mean	SD	CV (%)
CH_4_c (ppm)					
avg	7,110	114,574	401.8	179.1	44
npeaks	7,129	114,744	1.5	0.4	26
speaks	7,018	114,244	954.1	458.9	48
CH_4_p (g/d)					
Madsen	806	10,668	296.1	132.0	44
Chagunda	868	16,822	189.0	101.8	54
Kjeldsen2	798	10,676	309.3	135.1	44
Kjeldsen3	6,608	73,799	318.5	128.8	40
Tier2	855	14,635	355.1	68.1	19
Ratio	6,945	99,931	0.07	0.02	28
CO_2_c	7,529	141,591	5,376.0	1,846.0	34
Intensity (g CH_4_p/kg MY)					
MeI	6,608	73,799	9.9	4.8	48
Production trait					
MY (kg/d)	6,774	84,287	34.4	9.6	28
BW (kg)	868	16,868	697.6	82.7	12

The CH_4_p phenotypes were calculated according to the following formulas. Madsen was calculated as

CO_2_ (g/d) = 180 × 24 × (5.6 MBW + 22 ECM + 1.6 × 10^−5^ × number of days in gestation),

Chagunda was calculated as

CH_4_p (g/d) = mean CH_4_c/10^6^ μL × tidal volume (L/breath) × 30 breaths/min × 1,440 min/d × 16.04 (g/mol)/22.4 (L/mol),

where tidal volume (L) = 7.5 mL/kg of BW × BW (kg)/1,000 (mL/L). Kjeldsen2 was calculated as

CO_2_ (g/d) = −6134 + (213 × ECM) + (126 × MBW) + (52.5 × Milk CF) + (−5.13 × DIM) + 2117 + (−0.122 × DIM × Diet CF) + (0.386 × ECM × DIM) + (−1.18 × ECM × MBW) + (−0.614 × Milk CF × MBW) + (−5.96 × MBW) + (−1.18 × DIM) + (−0.614 × MBW),

where Milk CF is crude fat in milk, Diet CF is crude fat in the diet. Kjeldsen3 was calculated as

CO_2_ (g/d) = 8781 + (80.3 × ECM) + (−4.66 × DIM) + −49.0 + parity coefficient + breed-parity coefficient + (−0.149 × DIM × Diet CF) + (0.338 × ECM × DIM) + (206 × DIM) + (Milk CF-parity coefficient × Milk CF),

where parity coefficients (Kjeldsen et al., 2024) are 0 for first, 511 for second, and 1,587 for third and higher parities; breed-parity coefficients are for Holstein 0 for first, 775 for second, and 803 for third and higher; and MilkCF-parity coefficients are −4.18 for first, −10.5 for second, and −28.8 for third and higher parities. Tier2 (Equation 10.21; IPCC, 2019) was calculated as

CH_4_p (g/d) = (GE × 0.065/55.65) × 1,000,

GE = (NE_m_ + NE_a_ + NE_l_ + NE_P_)/REM/DE/100,

REM = 1.123 − (0.004092) × DE + 0.00001126 × (DE_2_ − 25.4/DE),

where gross energy (**GE**) was calculated with Equation 10.16 (IPCC, 2019), ignoring the net energy (**NE**) for growth, as we are using grown animals, and NE for wool, as it is not applicable. The NE_l_ = Milk × (1.47 + 0.4 × kg fat) and NE_m_ is net energy for maintenance, NE_a_ is net energy for activity, NE_l_ is net energy for lactation, REM (based on Equation 10.14, IPCC, 2019) is the ratio of net energy available in a diet for maintenance to digestible energy, DE is digestible energy as a percentage of GE, and CF is crude fat.

The Madsen and Kjeldsen formulas predicted CO_2_ (g/d), and this prediction was multiplied by the ratio of CH_4_c to CO_2_c to calculate CH_4_p. Variance components were estimated with a repeatability animal model in ASReml v. 4.2.1 (VSN International Ltd.). The model was as follows:

**y** = **Xb** + **Z**_a_**a** + **Z**_p_**pe** + **e**,

where **y** is the vector of phenotypes; **b** is the vector of fixed effects: herd-year-season interaction (n = 145), week of lactation (1–60), age of cow at calving (21–122 mo) nested within parity (1, 2, ≥3); **a** is the vector of direct additive genetic effects; **pe** is the vector of random permanent environment effects; and **e** is the vector of residual effects. The matrices **X**, **Z**_a_, and **Z**_p_ are the incidence matrices relating observations with the fixed effects, random genetic effects, and random permanent environment effects, respectively. Distributions of the random effects are var(**a**) = $A\sigma_{a}^{2},$ where **A** is the pedigree relationship matrix and $\sigma_{a}^{2}$ is the animal additive genetic variance; var(**pe**) = $I\sigma_{pe}^{2},$ where **I** is an identity matrix of an order equal to the number of observations and $\sigma_{pe}^{2}$ is the permanent environmental variance; and var(**e**) = $I\sigma_{e}^{2},$ where **I** is an identity matrix of an order equal to the number of observations and $\sigma_{e}^{2}$ is the residual variance. Pedigree included 64,334 animals with on average 14 generations. Bivariate analyses were conducted to estimate the genetic correlations among the different phenotypes. Due to the imbalance in record numbers of animals and records between traits, estimates of some pairwise bivariate analyses may be less precise and have reported large standard errors. Therefore, genetic parameters involving these traits (Madsen and Kjeldsen2) should be interpreted with caution.

Descriptive statistics for the different phenotypes are summarized in Table 1. The average CH_4_c phenotypes were 401.8 ppm for avg, 1.5 for npeaks, and 954.1 ppm for speaks. The average CH_4_p (g/d) varied depending on the formula, ranging from 189.0 ± 101.8 (Chagunda) to 355.1 ± 68.1 (Tier2). The average CH_4_p (g/d) was 296.1 ± 132.0 for Madsen, 309.3 ± 135.1 for Kjeldsen2, and 318.5 ± 128.8 for Kjeldsen3, indicating that these formulas predicted similar values for CH_4_p, which was expected as their formulas are the most similar. The average MeI was 9.9 g CH_4_p per kg of MY.

Estimated variance components are presented in Table 2. Heritabilities for CH_4_c phenotypes were 0.16 for avg, 0.29 for speaks, and 0.30 for npeaks. Heritabilities for average CH_4_c have been widely reported in the literature, ranging between 0.11 and 0.18 (López-Paredes et al., 2020; Manzanilla-Pech et al., 2022b; van Breukelen et al., 2023). Unlike these studies, Reintke et al. (2020) was the only study reporting heritability for the number of eructation peaks, which was 0.02 with an SE of 0.05. This study was conducted in ewes, and the lower heritability may be due to the method (laser methane detector) rather than the phenotype. Heritabilities for CH_4_p ranged from 0.03 (Madsen) to 0.27 (Tier2). The heritabilities for the ratio and MeI were 0.08, and was 0.13 for CO_2_c. Larger heritabilities (0.11–0.47) have been reported for CH_4_p using the Madsen formula by Manzanilla et al. (2022a,b) in a Danish Holstein population and by Sypniewski et al. (2021) in a Polish Holstein population. Genetic correlations between CH_4_c phenotypes (Figure 1) ranged from moderate between avg and npeaks (0.49, SE = 0.06) to highly positive between avg and speaks (0.85, SE = 0.02). As expected, the npeaks is moderately correlated with the 2 other CH_4_c phenotypes. However, because it does not reflect the intensity of the eructation, only the number, it is not a good candidate for a breeding goal trait. Conversely, speaks is highly correlated with avg, meaning that an animal with a high CH_4_c during the entire period also has a high value during the eructation peaks. Genetic correlations between CH_4_p phenotypes were highly positive (0.89–1.0), except for Tier2, indicating that despite numerical differences in the mean values, they rank the animals similarly (high and low emitters). Tier2 presented low to moderate (0.04–0.37) genetic correlations with the other CH_4_p phenotypes. Tier2 is used more as a control because it does not involve any input from the sniffers, but relies entirely on MY and BW. It has been developed to predict CH_4_p from a group of animals rather than individually (IPCC, 2019). However, it is important to mention that due to the low number of animals for some of the formulas that include BW in their calculation, some of the SE were high (0.06 to 0.30). Currently, there is no published information on the genetic correlations between the methane phenotypes presented in this study for either CH_4_c or CH_4_p, except for avg and CH_4_p with the Madsen formula. Genetic correlations between avg and CH_4_p were high (0.77–0.93), except for Tier2 (−0.08). Genetic correlations between speaks and CH_4_p were positive, ranging from 0.36 (Kjeldsen2) to 0.95 (Chagunda), and negative for Tier2 (−0.19). Similarly, npeaks correlations were positive, ranging from 0.52 (Kjeldsen3) to 0.99 (Kjeldsen2), and negative for Tier2 (−0.39). Genetic correlations between ratio and CH_4_c ranged from low negative (−0.18 to −0.08; speaks and avg) to moderate positive (0.59; npeaks). In contrast, the majority of genetic correlations between CH_4_p phenotypes and ratio were positive, ranging from 0.35 (Madsen) to 0.98 (Kjeldsen3), except for Tier2 (−0.25). No genetic correlation was estimated between CO_2_c and npeaks; however, moderate positive genetic correlations from 0.49 (CO_2_c and speaks) to high correlations of 0.84 (CO_2_c and avg) were estimated. Genetic correlations between CO_2_c and CH_4_p phenotypes were mostly moderate positive (0.18–0.58), except for Kjeldsen2 (−0.73), with a large SE (0.17).

**Table 2:** Estimated genetic $\left(\sigma_{a}^{2}\right),$ permanent environmental $\left(\sigma_{pe}^{2}\right),$ and phenotypic variance $\left(\sigma_{p}^{2}\right),$ along with h^2^ and repeatability (rep) with SE in parentheses for the average CH_4_c per visit (avg); sum of maximum peaks per minute (speaks); number of peaks per minute (npeaks); CH_4_p calculated by Madsen, Chagunda, Kjeldsen2, Kjeldsen3, and Tier2; carbon dioxide concentration per visit (CO_2_c); milk yield (MY); methane intensity (MeI); and BW

Trait	$\sigma_{a}^{2}$	$\sigma_{pe}^{2}$	$\sigma_{p}^{2}$	h^2^	rep
avg	3,540.4	6,049.40	21,979.0	0.16 (0.02)	0.43 (0.01)
speaks	49,496.8	37,027.6	171,040.0	0.29 (0.02)	0.51 (0.02)
npeaks	567.7	425.558	1,889.2	0.30 (0.02)	0.52 (0.02)
Madsen	245.1	2,585.2	8,369.8	0.03 (0.03)	0.34 (0.03)
Chagunda	1,670.0	2,548.9	7,914.0	0.21 (0.06)	0.53 (0.05)
Kjeldsen2	224.1	2,377.39	8,062.0	0.03 (0.03)	0.32 (0.03)
Kjeldsen3	707.6	1,504.6	8,465.7	0.08 (0.01)	0.26 (0.01)
Tier2	704.8	1,066.2	2,608.7	0.27 (0.07)	0.68 (0.06)
Ratio	0.28	0.46	3.53	0.08 (0.01)	0.21 (0.01)
CO_2_c	2,649.1	6,448.6	20,725.0	0.13 (0.02)	0.44 (0.01)
MY	12.2	22.1	54.6	0.22 (0.02)	0.62 (0.02)
MeI	1.1	3.1	13.5	0.08 (0.01)	0.31 (0.01)
BW	2,109.2	1,313.2	3,760.1	0.56 (0.08)	0.91 (0.08)

**Figure 1:**
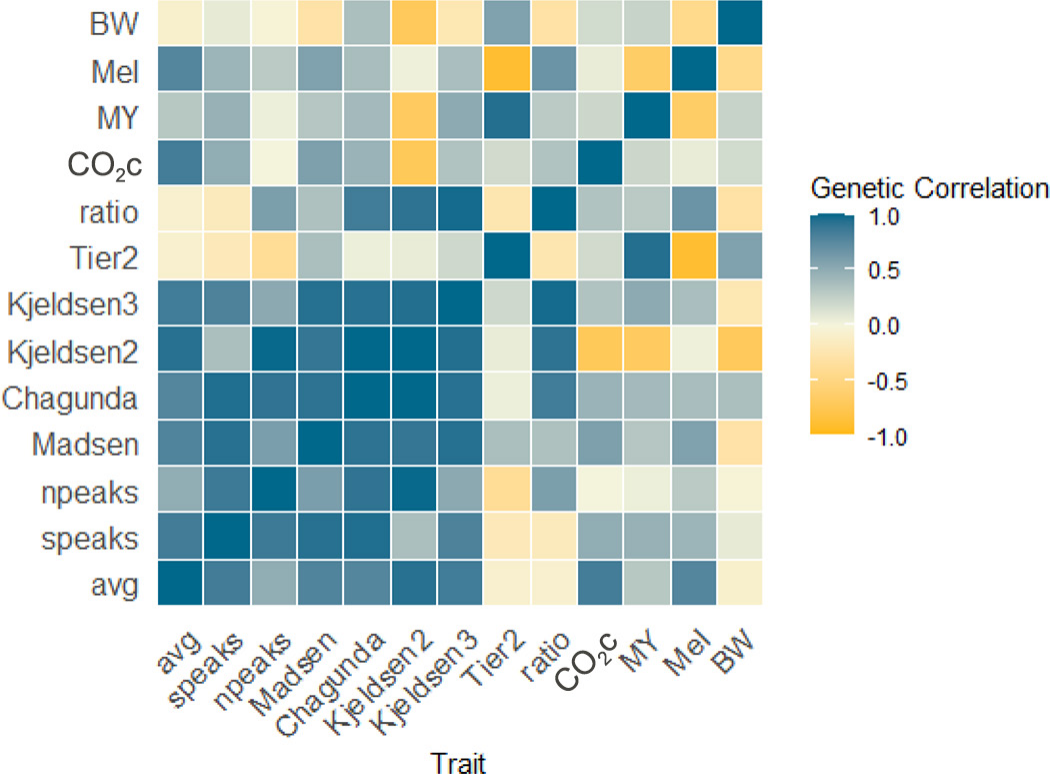
Genetic correlations among the average CH_4_c per visit (avg); sum of maximum peaks per minute (speaks); number of peaks per minute (npeaks); CH_4_p calculated by Madsen, Chagunda, Kjeldsen2, Kjeldsen3, and Tier2; carbon dioxide concentration per visit (CO_2_c); milk yield (MY); methane intensity (MeI); and BW.

Methane intensity presented the largest correlations with avg (0.77) and the smallest with npeaks (0.29), whereas for CH_4_p phenotypes, the largest positive correlation was with Madsen (0.57) and the smallest with Kjeldsen2 (0.03). However, because CH_4_p itself is derived from ECM alone or with BW, mathematical dependencies may artificially affect genetic correlations with MeI. Further, the genetic correlation between MeI and Tier2 was the largest negative (−0.90), which may also be affected by the shared use of MY in the calculation of both traits. Genetic correlations between MY and avg (0.30) and between MY and speaks (0.46) were moderately positive, whereas with npeaks, it was close to zero. This means that CH_4_c phenotypes such as avg and speaks are not entirely independent of milk yield. Most of the genetic correlations between MY and CH_4_p were positive (0.32–0.94), except for Kjeldsen2 (−0.69), with a large SE (0.97).

Finally, genetic correlations between BW and all CH_4_c phenotypes were close to zero, meaning that BW is independent of CH_4_c, as expected. For CH_4_p, the genetic correlations with BW were negative for Madsen (−0.30), Kjeldsen2 (−0.72), and Kjeldsen3 (−0.23), which can be explained by the indirect use of BW (in combination with ECM) in the prediction of CO_2_ for Madsen and Kjeldsen2. Positive genetic correlations between BW and Chagunda (0.36) and Tier2 (0.57) could be explained by the use of BW in the calculation of CH_4_p. Unlike the positive genetic correlation (0.65) between MeI and BW previously reported by Manzanilla-Pech et al. (2021) in an international dataset, in this study, this correlation was moderately negative (−0.45). One disadvantage of CH_4_p phenotypes using the formulas and MeI is the artificially created dependency on other traits that are used for their calculation, such as ECM and BW.

All phenotypes exhibited varying degrees of heritability. Average CH_4_c showed consistently high positive genetic correlations with all other methane phenotypes except for ratio and Tier 2 (benchmark). The CH_4_p phenotypes calculated using the formulas also presented strong positive correlations among themselves and with other methane traits. Given these results, avg and speaks appear to be good proxies for methane emissions, especially as they showed minimal correlation with body weight and no induced dependencies as the CH_4_p based on formulas. Overall, selecting for low-emitting animals is possible, regardless of the phenotype selected. The aim of this study is not to propose selection indicators, but rather to provide a fair comparison of different methane phenotypes that could potentially be used in future selection strategies for lower methane emissions when recording with sniffers. In this context, CH_4_c is considered the reference trait for methane emissions.
